# A systematic literature review of how and whether social media data can complement traditional survey data to study public opinion

**DOI:** 10.1007/s11042-022-12101-0

**Published:** 2022-02-14

**Authors:** Maud Reveilhac, Stephanie Steinmetz, Davide Morselli

**Affiliations:** 1 Lausanne University (Switzerland), Faculty of Social and Political Sciences, Institute of Social Sciences, Life Course and Social Inequality Research Centre, Lausanne, Switzerland; 2 Swiss Centre of Expertise in Life Course Research LIVES, Lausanne, Switzerland

**Keywords:** Social media, Survey data, Data complementarity, Public opinion

## Abstract

In this article, we review existing research on the complementarity of social media data and survey data for the study of public opinion. We start by situating our review in the extensive literature (N = 187) about the uses, challenges, and frameworks related to the use of social media for studying public opinion. Based on 187 relevant articles (141 empirical and 46 theoretical) - we identify within the 141 empircal ones six main research approaches concerning the complementarity of both data sources. Results show that the biggest share of the research has focused on how social media can be used to confirm survey findings, especially for election predictions. The main contribution of our review is to detail and classify other growing complementarity approaches, such as comparing both data sources on a given phenomenon, using survey measures as a proxy in social media research, enriching surveys with SMD, recruiting individuals on social media to conduct a second survey phase, and generating new insight on “old” or “under-investigated” topics or theories using SMD. We discuss the advantages and disadvantages associated with each of these approaches in relation to four main research purposes, namely the improvement of validity, sustainability, reliability, and interpretability. We conclude by discussing some limitations of our study and highlighting future paths for research.

## Introduction

This paper provides a systematic literature review of how social media data (SMD) and traditional survey data have been used complementarily to study public opinion (PO) over the last decade. As social media users represent more than half of the world’s population (see [26]) and provide continuous reactions to daily socio-political events, it is not surprising that traditional survey research has been concerned about whether such data would make surveys obsolete or whether they could be used complementarily. Addressing these questions is particularly relevant in the area of PO. Social media plays a growing role in the formation of PO as user-generated content on these platforms is increasingly deployed as representations of PO (e.g. [27, 56]). In addition, politicians increasingly consider social media, especially Twitter, to be a “barometer” of PO [44].

Despite the extensive literature about the benefits and challenges of using SMD to answer social and political questions, as well as about SMD as a possible replacement for traditional surveys, a comprehensive overview of the complementarity of both data sources remains limited. The aim of this paper is to fill this gap by providing a systematic literature review focusing on how SMD and survey data can complement each other to study PO. Inspired by the influential study of Japec et al. [45] which elaborated on the complementarity of survey data and “big data” (rather broadly defined), we want to concentrate, however, on one type of “big data”, namely SMD. There are two main reasons for this choice. First, SMD are a specific type of “non-survey” data which possess specific arrangements (or conventions) and paradata that are different from other types of administrative or “big data”, especially when it come to the assessment of PO. Second, whereas there is substantial research on augmenting survey data with administrative (e.g. electricity or water consumption) or other type of “web data” (e.g. Google searches or citation metrics) to improve estimates of PO or official statistics, we still lack an overarching picture of the (new) developments and approaches of complementing SMD and surveys with each other.

Our analysis is based on an extensive survey of the literature capturing a representative sample of the best published theoretical and empirical scientific papers on the topic (N = 187). We have restricted the analytical period to the last decade (2010–2020) as the discussion on complementarity is still a young field of study (e.g. [58]). On this basis, we have been able to identify six complementarity approaches which can be synthesised to four major purposes, namely predicting, substituting, comparing, and linking SMD and survey data.

In the next section, we situate our review within the existing literature by demonstrating how the scientific discussion surrounding the opportunities and challenges offered by SMD within survey research has evolved, especially by highlighting the complementary understanding of PO offered by both data sources. Then, we discuss more specifically which research approaches have emerged, and we classify them according to four main research purposes using both data sources complementarily. The analysis of the empirical studies aims to act as a guide for other researchers by identifying research gaps and highlighting the pros and cons of each approach. Furthermore, we underline areas for future improvements and point to technical and ethical considerations. We conclude by mentioning the main contributions and limitations of our review.

## Background – The complementary understandings of PO

Surveys have long been the most predictive and accurate tools for collecting and measuring opinion. However, over the last decade, decreasing response rates have called into question the potential of using a random sample of individuals to represent an entire population (e.g. [37, 49]), thus posing important concerns about the sustainability of survey research. Even by adapting to new modes, such as push-to-web, to increase response rates, it remains unclear whether surveys will maintain this dominant role as communication habits continue to change (e.g. [68]). Given the recent “survey crisis” (e.g. [13, 22]), an increasingly rich source of PO data is commonly referred to as “big data”. These “new” data take the form of extraordinarily large and complex datasets. There are three attributes that are generally agreed upon to describe this type of data (e.g. [19]), namely volume, velocity, and variety. Social media are a sub-type of big data where people express their thoughts and opinions with the purpose of sharing them with others [18]. Due to their inherent properties, SMD have been seen as a promising complementary, and even alternative, source of data for exploring PO. However, researchers acknowledged early on that, almost universally, SMD are non-random, and thus discouraged using them as a means of making generalisable claims. This challenge is well highlighted by Schober et al. [68], who claim that, while the social media researcher seeks to achieve topic coverage, the survey researcher emphasises population coverage as a central endeavour.

An entire strand of research thus focussed on how surveys and social media differ in several aspects. Table 1 attempts to classify the most prominent differences along which SMD and survey data are typically compared. We have identified several dimensions based on recurring criteria mentioned in the literature concerning the nature of and the relationship between both data sources. Often-cited criteria include the type of population and data signal, the unit of observation and analysis, and the available meta-data (for a thorough discussion of the differences see [18, 68], and [77]).

**Table 1:** Differences between SMD and survey data to study PO

	SM	Survey
Type of population	Selective	Representative
Data signals	Platform users and their signals (e.g. posts or tweets, #hashtags, @mentions, retweets, replies)	Opinion survey of individuals with a defined sampling frame
Types of data	Unstructured texts containing opinions and opinion strength or merely information (e.g. links)	Structured opinions measured by answers to pre-defined and pre-tested survey questions (scales)
Unit of observation or the level at which data are collected	Can be any of the following: users, search queries or keywords, #hashtags or @mentions, retweets, or replies, likes or emotional reactions, location	Individuals from a sampling frame representing a target population
Unit of analysis or level at which the data are analysed	Can be any of the following: users, location, texts from a specific topic or sentiment, overall texts, links, or other metadata	Individuals’ responses to survey items or aggregated responses at the country, region or household level
Meta-data	Set of users’ behavioural information (e.g. network, frequency of use, interactions) and contextual information (e.g. time and location)	Precise and quasi-complete socio-demographic information on individuals and auxiliary data (e.g. number of contact attempts, number of persons in the household)

To understand how to best use both data sources complementarily, it is also essential to reflect on how they construct PO differently. This is increasingly important, as what constitutes “the public” tends to be forged by the methods and data from which it is derived [56]. In survey research, PO is equivalent to the private opinion of a representative public, operationalised as a set of positions on a given topic. PO can thus be conceptualised as a reflection of a shared position among citizens on specific issues that are then amplified and reviewed by news media and political actors [42]. Survey measures of PO are constrained by the scope of the questionnaires, which usually provide little room for spontaneous expressions of opinion (except in open-ended survey questions). The diversity of opinions is thereby reduced into a set of discrete and aggregate data (e.g. [75]). Conversely, the reliance on social media for measuring PO expands the societal and collective components of opinions [59] by conceptualising it in Habermas’ [39] terms as a complex system of representations. In this respect, SMD are better suited to capturing the conversational and relational nature of PO formation [3]. Hence, where survey data weigh precision and standardisation, SMD excel in multidimensionality and polyphony. In addition to their focus on solicited private opinions, surveys are also less reactive to opinion changes than SMD. In theory, opinion changes could be assessed by frequent short opinion surveys (e.g. every two months). However, the advantage of SMD is that they can cover opinion change more rapidly (and on an ad hoc basis), thus reacting faster to events, which is almost impossible for surveys (e.g. it takes more time to set up probability-based surveys for the study of COVID compared to what can be done with SMD).

Despite the advantages offered by social media for measuring more social and timelier opinions, the reliance on SMD raises important questions for empirical research on (automated) measurements of opinions and on the choice of the indicators employed to model opinions. Indeed, constructing measures of PO based on SMD can be very time consuming and can involve a lot of pre-processing effort before the data can be translated into meaningful measures of expressed opinions. Furthermore, it sometimes remains quite difficult to know what is driving the evolution of ideas and concerns found in online conversations. Consequently, a current strand of research seeks to better understand the issues of representativeness of social media communities and the validity of measured opinion, especially opinions stemming from sentiment analysis. While there is a rising interest in applying SMD to understand opinion, and even to replace traditional surveys (e.g. [3, 32]), SMD alone are of limited use for social scientific research as they usually provide incomplete and imprecise information. However, the issues associated with SMD are not necessarily fatal to the proposition that they can be used to generate social insights, especially in complementing survey data. An efficient strategy to enhance research lies, therefore, in the analysis of how both data sources can complement each other in ways that maximise their strengths.

In the next sections, we aim to show that there is a plethora of research practices in which both data sources complement each other for the study of PO. To date, however, there is still no consensus about the best way to use SMD for studying PO [58]. We are now at a point where we should reflect on what has been done so far, what lessons we can learn from it, and then specify suitable trends for social research. In this paper, we seek to fill this gap by reviewing research that uses both data sources complementarily for the purposes of measuring PO and by providing a critical evaluation of the identified research paths.

## Method of analysis: Building a corpus of relevant articles

To build our corpus of scientific articles, we carried out several searches in bibliographic databases (focusing on *Scopus* and *Google Scholar*) using the software *PublishOrPerish* [40]. We obtained an initial corpus of 3596 unique papers, which we reduced to papers that were relevant for the scope of our review. The initial corpus was deliberately based on a search-query that was broad enough to collect the relevant literature, while not missing important papers. We used the query “(social media OR twitter OR facebook OR instagram OR reddit) AND (survey OR surveys OR polls)” and specified that it should appear in the body of the text (using the *keyword* field) instead of appearing only in the title or abstract, which were found to be too restrictive to capture the literature of interest. The query was designed to restrict the focus of our review to SMD, thus ignoring other types of “big data” or “digital trace” data.

A first filter was applied to reduce the number of papers to journal articles, book chapters, and scientific reports (thus excluding books, theses, and conference papers) as we wanted to concentrate on high-valued scientific sources which have already been approved by the scientific community. In this respect, including conference papers would have drastically inflated the number of (duplicated) papers concerned with predictions and with replicating previous studies using alternative methods of analysis and algorithms. Among the remaining papers, we applied two eligibility criteria to disregard those that were not pertinent to the analysis as i) their focus was not on PO, ii) they were oriented towards a specific aspect of data treatment (e.g. estimating socio-demographics from texts or profile pictures) or an analytical strategy (e.g. elaborating algorithms). We also excluded articles mentioning survey findings without an explicit aim of supplementing, comparing, or combining those with SMD.

## Results of the literature review on the uses of social media as a complement to surveys

**Fig. 1:**
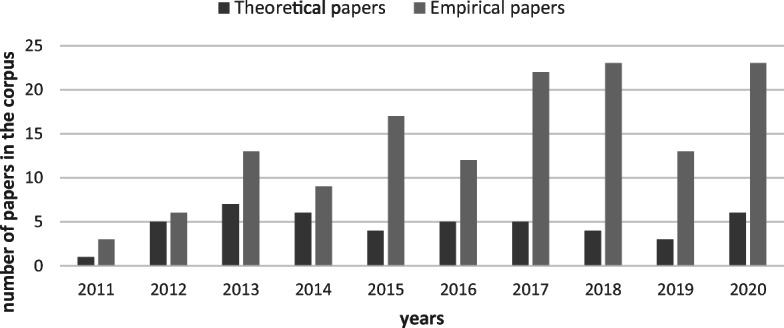
Number of empirical and theoretical articles according to our meta-review of the existing literature using surveys and SMD

Overall, the collection protocol left us with 187 papers - 141 of an empirical and 46 of a theoretical nature (these papers can be found in the Appendix). Most of these papers stem from political communication and computational social sciences journals. Although the sample of 187 papers may not cover the whole corpus of research on the subject, it is nonetheless sufficient to highlight the main research directions that have been endorsed on the topic of complementarity. Figure 1 provides an overview of the yearly repartition of the retrieved papers differentiating between those with a theoretical (N = 46) and an empirical (N = 141) focus. While the number of theoretical papers remains stable over the years, we can see a steady increase in empirical papers over time.

### Theoretical insights

Starting with the *theoretical papers* in our review (N = 46, see Table 2 in the Appendix), survey and social media researchers have explored ways in which social media and survey data can yield congruent conclusions (e.g. [68]). One part of these articles (n = 14) tries to establish a framework regarding the predictive power of SMD as a potential substitute for surveys. This line of research stems principally from the fields of election and economy forecasting (for recent reviews see [15, 66]).

Another strand of theoretical articles (n = 14) focuses instead on the compliance of social media research with established reporting standards so as to guarantee transparency and replicability (e.g. [51]). Finding ways of integrating data obtained from different sources (n = 3) also constitutes a fertile path of research [46]. In this respect, Stier et al. [72] provide the most advanced guide on how to systematically link survey data with information from external data sources, including SMD, at different level of analysis. The authors demonstrate that integrating traditional survey data and digital trace data is of growing interest, notably because of the limited reliability of self-reported behavioural measures and declining response rates. Additionally, enriching survey data with SMD could also help to reduce unit non-response and to control for the unrepresentativeness of SMD, as they are limited to those respondents having social media profiles and consenting to the linkage. Finally, a smaller share of research (n = 5) focuses on developing a quality assessment framework for SMD which is similar to the *Total Survey Error* (TSE) [11, 38]. The TSE framework has been extended to encompass SMD and their inherent quality challenges (see the studies by Sen et al. [70] on Twitter-based studies and Jungherr [47] for a measurement theory to account for the pitfalls of digital traces). In a similar vein, Hsieh and Murphy [43] analysed the potential benefits of evaluating estimates from surveys and SMD in common terms and arrived at a general error framework for Twitter opinion research. Olteanu et al. [61] went a step further by pointing to the errors and biases that could potentially affect studies based on digital behavioural data, outlining them in an idealised study framework. The paper by Sen et al. [70] provides the most advanced framework to date. It involves potential measurement and representation errors in a digital trace-based study lifecycle where they are classified according to their sources.

Other research (n = 5) tackles the ontology of SMD as compared to survey data. In these papers, prevalent discussions revolve around the conception of opinion as measured by both data sources, as well as debates related to the evolution of “new” research “paradigms” or “digital hermeneutics”. The remaining papers concentrate on behavioural research (n = 2), demographic research (n = 2), and small data analysis in political communication (n = 1).

Overall, the considered theoretical articles stress the importance of developing a framework that accounts for possible biases of SMD while remaining in, or mirroring, the TSE. Moreover, they also emphasize the need, in this debate, to focus on the complementarity rather than the replacing aspect, notably by developing clear and reliable linking strategies. These articles also encourage researchers to go beyond the dominant model for understanding PO from probability sample surveys to encompass other (“new”) expressions of opinions (e.g. Murphy et al. 2014) that can possibly supplement or even replace survey-based approaches.

### Empirical insights

The *empirical literature* (N = 141) focuses on a rather narrow set of topics, such as elections, political issues, and approval ratings for the presidency (64%). Another important area of PO research using SMD complementarily with survey data is related to health (e.g. vaccination, drugs, etc.), equality issues, and climate or environment-related concerns. Most empirical studies in our review are based on Twitter data (73%), followed by Facebook (18%) and other social media (9%). This is related to the fact that not all social media platforms provide the same degree of data accessibility [8]. For instance, Facebook imposes severe limitations on the scope of retrievable data, whereas Twitter has less strong privacy settings, allowing researchers to get access to Twitter’s historical data.

**Fig. 2:**
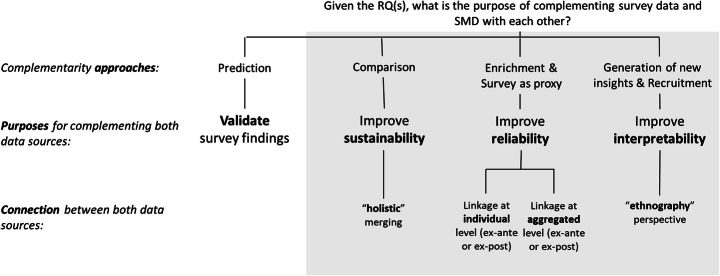
Complementary approaches using SMD and survey data for the study of PO

Overall, we derived six major approaches on how survey data and SMD can complement each other namely i) predicting social and political outcomes using SMD (n = 48), ii) comparing both data sources on a given phenomenon (n = 26), iii) using survey measures as a proxy in social media research (n = 18), iv) enriching surveys with SMD (n = 9), v) recruiting individuals on social media to conduct a second survey phase (n = 8), and vi) generating new insight on “old” or “under-investigated” topics or theories using SMD (n = 32). These approaches can be synthesised in four, partly overlapping, ‘data complementing’ research purposes: i) validating survey findings with SMD, ii) improving the sustainability of the research by diversifying the views on a phenomenon, iii) improving the reliability of survey measures by specifying measurements, and iv) improving the interpretability of social or political issues. Figure 2 summarises the relationship between the six approaches and the four research purposes. Furthermore, it shows that each purpose leads to a typical way of using both data sources complementarily. For instance, improving reliability by specifying a research question involves data linkage strategies, while generating new insights involves a sequential use of social media and survey stages.

The analysis of our corpus suggests that the biggest part of research concentrates on whether SMD can potentially substitute survey data (n = 48, see Table 3 in the Appendix). This has mostly been done by trying to replicate survey findings by using SMD for forecasting (see recent review by [66]). The aim to predict real-world outcomes with SMD in the realm of PO has essentially been applied to elections. Most of these papers directly refer to the much-cited study of O’Connor et al. [60] which purpose is to validate SMD against survey findings. While research in this area has tested a range of different methodologies, the results remain inconclusive, and only in some cases could elections be accurately predicted (e.g. [31, 47]). Recent literature reviews on the use of SMD for running electoral predictions (e.g. [15]) classify studies according to the employed methods of prediction, such as volume, sentiment, or network approaches. These reviews show considerable variance in the accuracy of predictions, which, on average, lag behind the established survey measurements. A common problem of the aforementioned studies lies in the decision about which approach can most accurately yield predictions (but also which social media platforms are better suited, and how that varies in different geographical or temporal contexts). This inference problem is quite complex as various elements are involved in skewing the samples in social media debates. To date, the inconclusive state of the research has led to a research agenda aiming to respond to the plea from Gayo-Avello et al. [33] for a “model explaining the predictive power of social media” (p. 490). In this realm, for instance, the study of Pasek et al. (2019) assesses how patterns of approval among population subgroups compare to tweets about the president, while disentangling effects at the individual and group levels of analysis. On a more theoretical level, the study by Schober et al. [69] seeks to elaborate when and under what conditions SMD can be used to make valid inferences. However, the inconclusive state of the research may also be linked to the fact that predictions are often done based on the content created by users and overlook the characteristics of the creating users. For instance, SMD can be biased towards a particular group (see [5, 24]). Moreover, interactions on social media platforms are not always the product of individuals, but also bots, organisations, political parties, etc. [80]. Based on the evaluation of the body of articles falling under the ‘substitution paradigm’, a path for future research could be to better account for the characteristics of social media users, insofar as these characteristics can be useful for assessing how individual tweets can be converted into meaningful measures of expressed opinion. To do so, future studies could survey social media users identified using relevant key terms (e.g. hashtags or mentions) to gauge the relationship between social media measures of their sentiment and survey measures of their attitudes.

The second dominant approach in our review is related to how surveys can be enriched with SMD (n = 9, see Table 4 in the Appendix). Here, SMD are collected with the intention of improving the reliability of survey measures at the individual or aggregate level. Replication of survey-based opinions can be difficult, either because of improper interpretation of the findings or because insufficient information has been provided. Such issues undermine the credibility of survey research and make it difficult to evaluate the contributions of a given study. Research aiming to enrich surveys with SMD most often implies the adoption of a data-linking strategy. This can be done, for instance, either at the user level, public actor level, geographic level, or temporal level (see [72]). Enriching surveys with SMD can serve several goals. First, it can help to augment the explanatory potential of survey measures. For instance, De Sio & Weber [23] adopted an innovative research design to explain election outcomes based on party strategy on social media with respect to policy issue salience. They did this by linking representative mass surveys from six European countries with Twitter analysis of campaign activity. Second, enrichment of survey data with SMD can also help to test research hypotheses by relying on “true” behavioural measures (instead of self-reported survey measures). For instance, Karlsen and Enjolras [48] linked candidate survey data with Twitter data to study styles of social media campaigning. These differences in campaigning styles were then related to the extent to which candidates were successful on Twitter. Third, SMD also offer an opportunity to address issues of item non-response and calibration of novel measures. For instance, Shin [71] studied the extent to which social media users selectively consumed like-minded news stories by linking survey responses from Twitter users with their media following and exposure to news via their friends. The study further showed some differences between self-reports and digital measures, such as more pronounced patterns of selective exposure in the SMD. Finally, linking social survey and SMD further provides an opportunity to explore the relationship between attitudes and beliefs reported through surveys and content (and behaviours) generated online. For instance, Cardenal et al. [14] combined survey and Web-tracking data to analyse how Facebook-referred news consumption influenced social media users’ agendas. They found that selective exposure increased with amplified news consumption. The core problem in these studies lies in gaining consent to carry out the data linkage. This constitutes a complex procedure in which issues of anonymity, security, and disclosure all come to the fore. An additional problem is that social media measurements provide only one partial view of opinions. For instance, while researchers can measure how many times a given message has been liked, shared, or retweeted, it is much harder to account for (or measure) how often a given message has been seen or has attracted attention. Moreover, our corpus shows that research relying on linking strategies tends to remain at the individual and public actor levels of analysis, which requires requesting consent to use the linked data. This may, in turn, introduce consent or selection bias. To mitigate such difficulties, future studies should also explore the potentials of linking both data sources at higher levels of analysis, such as country or according to topicality level.

A third purpose is to use surveys as a proxy in social media research. This approach therefore reverses the logic that SMD are always used as a complementary (side) element of the main survey-based analyses. In this kind of “survey proxy approach” (n = 18, see Table 5 in the Appendix), SMD are used as the main source of analysis, while the survey data are used for contextualising or calibrating SMD. A first strand of research relies on SMD to complement traditional research approaches in political communication and citizens’ political engagement. For instance, the assessment of the importance of given public concerns in PO has been measured extensively with the “most important problem” survey item. Social media provide another way to measure this concern in an unintrusive way by (semi-)automatically classifying the content of social media texts, while also accounting for the extent to which different actors are responsive to these concerns. Following this logic, the study conducted by Eberl et al. [28] investigated the effects of sentiment and issue salience on emotionally labelled responses to posts written by political actors on Facebook. Another study, by Plescia et al. [64], analysed the responsiveness of populist parties to the issue salience amongst the public. They did this by relying on survey data to measure public salience and tweets to assess salience issue for parties. A second strand of studies aims at facilitating cross-national comparisons. For instance, a possible application consists in using survey data for classifying parties and voters along important dimensions (e.g. see [30]). Here, parties were placed on a left and right spectrum using the Chapel Hill Expert Survey [4]. Party score on the overall ideological stance was then used as an explanatory variable in subsequent analysis. Another example is the study by Park et al. [62] which investigated the consumption of popular YouTube videos in countries that differ in cultural values, language, gross domestic product, and Internet penetration rate. A possible issue encountered by these studies is linked to spurious effects between survey and social media measurements (e.g. misleading or unexplained correlations). Furthermore, these studies tend to remain poorly equipped to explain actual motives behind social media users’ expression of opinions or reactions. The “survey as proxy” approach requires a considerable dose of ingenuity and methodological innovation to mine social media for producing opinion estimates that can be merged with survey estimates. For instance, SMD corpora often deviate from a predefined (survey) coding scheme. Substantively, a future path of research should take advantage of the fact that a growing number of societal issues have become transnational, such as immigration, terrorism, women’s rights, and climate change. Such research could involve the combination of word embeddings and survey opinion measures at the country level.

A fourth approach aims to compare SMD with survey responses that directly measure PO. Studies comparing SMD with survey data (n = 26, see Table 6 in Appendix) essentially aim at improving sustainability of the research, which consists in the ability to gauge PO consistently over time. Sustainability thus implies that we should develop designs that include opportunities for “holistic merging” of the data that will generate more inclusive and fine-grained research insights. There are several reasons that comparing both data sources is meaningful for social research. Firstly, comparing SMD and survey data can be very useful in times of protests and collective actions, notably due to the difficulty of generating survey data to properly assess these disruptive changes (see critique of survey data by Lee [53]). The timing of an event might indeed not coincide with the timing of a survey, which is often done ex-post. For instance, Davis et al. [21] examined the extent to which tweets about the affordable care act (“Obamacare”) could be used to measure PO over time. Secondly, social media can be compared to surveys for research questions that require chronicity, on a weekly or daily basis, thus going beyond the few ongoing surveys that collect data monthly or yearly. For instance, Diaz et al. [25] demonstrated how social media activity functions like an “opt-in panel” where users repeatedly discuss the same topics. This allows us to study, longitudinally, quite rapid shifts in individual opinions and behaviours, thus complementing survey panels which are prohibitively expensive. Another example is the study by Loureiro & Alló [54], which aimed to complement surveys by providing up-to-date measurements about social concerns when debating mitigation and energy transition paths. Thirdly, survey questions are often designed to capture internal attitudes toward a specific object. However, the relevance of certain survey questions might vary over time and, in some cases, might no longer correspond to the issues discussed spontaneously online. For instance, at a geographical level, the study by Scarborough [67] compared gender equality attitudes found in survey data to sentiments emanating from tweets. Fourth, SMD can produce quicker and less expensive statistics for enabling informed policy and program decisions. However, this requires gaining knowledge of where any possible disparities in attitude distributions between SMD and survey data may lie. In this respect, the study by Amaya et al. [2] presented recent advancements. The authors compared attitude distributions between Reddit users and survey measures of political leaning, political interest, and policy issues. They showed that Reddit users tend to have more centrist and normally distributed scores than the survey data, skewing estimates toward the conservative end of the spectrum on all attitude measures. Another study, from Pasek et al. (2020), explained that SMD might be better conceived as providing insights about public attention rather than (“survey like”) attitudes or opinions. To do so, the authors compared tweets mentioning the presidential candidates and open-ended survey questions about the candidates to assess whether spikes surrounding political events correlate between both data sources. Results display some support for the correlation between social media attention and survey data, but they also show systematic differences that need to be better understood to assess when SMD can best generate insights about select topics. The research comparing both data sources tends to remain focused on volume analysis and tonality assessment. This type of research also tends to pay little attention to the domain-specificity of the SMD collected as well as to ways of mitigating replicability and consistency issues (e.g. [34]). For instance, the evolution of search queries around a given theme might lack precision and consistency over time. The connotation of hashtags can change or whole hashtags can even disappear. Better combining both data sources also requires elaborating more sophisticated measures of opinion and attitudes. One could think about pushing forward “stance detection” in complement to “sentiment detection”, but also about advancing “narrative analysis” in complement to “topic or frame detection”. These are avenues where computational social research would benefit from the expertise of applied computational linguistics.

A fifth approach implicates using SMD to generate new insights. This is especially useful when survey data are not available or when survey data are not recent enough (n = 32, see Table 7 in Appendix). Here, the main purpose is to improve the interpretability of the research by adopting an “ethnographic” methodology. By avoiding rigid research design plans, SMD can remain responsive to, and pursue, new paths of discovery as they emerge. Based on the papers collected, we found typical reasons for relying on SMD to generate new insights, such as capturing emergent opinions, expanding the scope of survey measures, validating survey measures, proposing novel approaches to get a more nuanced or dynamic perspective on PO, and making causal analyses (see column “Reason to complement” in Table 6 in Appendix). When used for capturing emergent opinions, SMD allow us to study the topical and normative climate around specific issues for which we have no theoretically grounded ideas yet. In this exploratory design, social media can provide survey researchers with a snapshot of important societal and political concerns worth surveying in future research. This is especially useful for emerging topics, such as nuclear power (e.g. [50]) or health-related policies [65, 74]. On these emerging issues, SMD can be used in an exploratory or ethnographic perspective to generate initial and qualitative insights into under-studied research objects in order to develop quantitative survey measurements. SMD can also be useful for expanding the scope of survey measures on topics that are difficult to survey. For instance, Hatipoğlu et al. [41] used SMD to study international relationships with a case study on Turkish sentiments towards Syrian refugees using Twitter. Another study by Guan et al. (2020) relied on the social media platform Weibo to study Chinese views of the United States. SMD can also be useful for validating survey measures. For instance, the study by Dahlberg et al. [20] investigated the meanings of democracy in a cross-country perspective to better understand differences in the usage of the term “democracy” across languages and countries. The authors’ findings aimed to inform survey measurements about the different conceptualisations of democracy, notably by highlighting translations and language equivalence issues in survey items. Another reason is to propose novel approaches for achieving a more nuanced or dynamic perspective on PO. For instance, researchers can add new components and improve “old findings”, which are difficult to measure with survey data. In this view, the study by Barberá et al. [7] modelled policy issue responsiveness using Twitter data, thus going beyond the more static perspective on issue congruence offered by surveys. In another study, Clark et al. [16] investigated organisational legitimacy in a case study about public reactions on social media to the Supreme Court’s same-sex marriage cases. The authors argued that SMD can lessen some of the limitations of survey research in the field, notably by accessing not just policy positioning among individuals but also a variety of features of political discourse, such as opinion intensity and emotions like anger or happiness. SMD can also be used to make causal inferences in order to understand changes in opinion before and after an event, such as measuring the effect of a promulgated law on PO [1]. Here, SMD allow researchers to rely on spontaneous opinions expressed online rather than on retrospective survey questions, and this can help develop policy initiatives. For instance, Tavoschi et al. [73] used Twitter as a “sentinel system” to assess the orientation of PO in relation to vaccination. Despite the advantages of SMD in providing new research insights, these studies tend to lack a rigorous contextualisation of the findings derived from SMD. In this respect, a reliance on SMD would benefit from implementing sequential designs, where social media help to identify specific populations or sub-topics, which could then lead to a second quantitative survey phase. Whenever possible, SMD would further benefit from a comparison with longitudinal surveys to assess the extent to which both data sources reveal similar dynamics of change. Future studies could further exploit SMD’s ability to generate new insights for research in sensitive fields, such as war, racism, sexual orientation, and religious beliefs. These are often topics on which it remains difficult to collect survey data, notably because of the social desirability bias (e.g. [52]) and the like (e.g. extreme response style, moderacy bias, and acquiescence), but also because of the fear of being denounced or because the topic is controversial.

The last approach using SMD and survey data complementarily focuses on using social media to recruit survey respondents. However, in comparison with the previous approach, the studies collected here usually analyse SMD and survey data in sequential phases. As we only consider papers that are in some way also related to PO and are not solely about recruitment of survey respondents and their socio-demographic characteristics, the number of studies we were able to analyse is much smaller (n = 8, see Table 8 in the Appendix). Our review demonstrates that the papers essentially tackle the problem surveys have in recruiting specific politically involved sub-groups of the population. In particular, the research relies on social media to access representative samples of social media users, for instance, those who commented on their countries’ elections (see [9, 12]) or who posted at least one election-related tweet [79]. Furthermore, in these studies, ethical concerns (e.g. privacy, tracking, etc.), but also the technical affordability of the social media platform used, are discussed. The latter issue is important, as each social media platform has particular arrangements which are likely to influence the group of individuals that can be reached. Overall, future studies could think about extending the recruitment approach to enhance our knowledge of reactions to systematic events, topics, or other repetitive features (such as supporting an issue or taking part in actions), while eliminating recall errors. Furthermore, relying on SMD can help researchers pre-test their hypotheses for future surveys by uncovering relevant underlying discursive patterns or by making smaller-scale qualitative observations.

## Summary and concluding remarks

The aim of this article was to provide a review of published papers on the complementarity of SMD and survey data for PO research. We started this review by situating our work within theoretical advances concerning the complementarity of both data source. There has been extensive work underlying the opportunities and (quality) challenges of SMD for answering social research questions. However, research attention has only recently turned to SMD as a source of expression of PO and of its measurement. Consequently, there is a need for more research to uncover the ways in which SMD can be best used for fostering the understanding of PO.

The main contribution of our review is to provide a complete picture of the empirical research on the topic while calling attention to the pros and cons of each approach and possible future paths of advancements. Though this review might not be exhaustive, it has enabled us to show six major complementarity approaches which were identified as responding to four different research purposes. Below we highlight the main research paths for each approach. Using both data sources complementarily for prediction purposes was by far the most prominent approach and it remains a research area which raises many questions about the potential generalisability of the findings, namely in terms of the representativeness and validity of social media measurements of PO. We believe that the most important difficulty lies perhaps in the manner in which these studies deduce political opinions or attitudes from SMD. Survey researchers readily admit that opinions are more difficult to measure than behaviour because they involve what people think and not just how they act. Thereby, the choice to rely on sentiment analysis or merely on volume metrics (such as the number of retweets or mentions) seems unclear, at least for the near future.

Approaches concerned with improving sustainability have a significant potential for advancing social research, as they allow researchers to combine the richness of SMD content with established survey measures. When SMD are used in similar contexts to survey data, we believe that a critical view should prevail, informed by current social science best practices and expertise. For instance, whereas surveys draw a sample of carefully worded and standardised questions, social media can cover many topics as well as different facets of the same topic, which are not necessarily defined a priori on a theoretical basis. This research avenue is most likely to be fruitful for studies aiming to augment surveys by mapping discussions that are topical on social media, while allowing variations at country or regional levels of analysis to be discerned (e.g. Bennett et al. [10] on climate change opinions). Studies aiming to compare both data sources are certainly the most suitable to help improve our understanding about when and how both data sources can be validly combined. Survey methodologists can play a decisive role, notably by paying attention to the type of (open-ended) questions that can be more directly comparable with SMD. This direction can also inform the lack of consistent evidence for the first prediction approach.

Alternatively, studies aiming to improve reliability see research as mostly requiring control for the still severe limitations of using SMD appropriately in a PO context. In this respect, studies enriching survey data with SMD offer a solution to the fact that social media often lack relevant individual information, such as respondent’s attributes (e.g. sociodemographic characteristics or personality traits) or key outcome variables (e.g. voting, social, or political attitudes). Additionally, the “survey as proxy” approach enables researchers to calibrate SMD according to standardized survey measures at the actor (e.g. political candidates or parties) or context levels by reversing the data linking strategy. Future paths for both approaches implicate opening up the analysis to non-individual levels.

Studies aiming to improve the interpretability of survey research by generating new insights or by recruiting respondents on social media for a second survey phase, and that use both data sources complementarily, offer additional fertile ways to consider for new analyses that would not be possible using survey data alone. In this view, SMD do not aim to replace opinion surveys, but aim to provide a broader context for interpreting opinion, which will then serve to improve the quality of survey questions. This research avenue is most likely to be useful for knowing more about hard-to-reach populations (e.g. the LGBTQI* or disabled persons communities) or topics that are difficult to survey (e.g. violence and racism), especially when conducting iterative phases of analysis. It is also useful to get “opinion climates” about topics which have long been under survey scrutiny (e.g. emerging concerns related to feminism or social inclusion) in order to develop “updated” survey measurements.

Bringing together the opportunities offered by these different approaches shows that samples of social media users do not necessarily have to be representative of the general public to be used meaningfully as a complement to surveys. Most importantly, we believe that SMD should supplement, but not replace, traditional methods and data sources in the study of PO. By keeping up with current developments, we believe that remaining in the framework of survey research when using both data sources complementarily is paramount for identifying potential non-survey data sources, accessing them, and assessing their quality and usefulness for the study of PO. Like mixed-method approaches combining qualitative and quantitative data (e.g. [36]), the primary motive for complementing survey and SMD with one another is to allow researchers to mix datasets in a meaningful way for developing an overall interpretation.

### Technical and ethical note

Regarding sustainability, it is important to consider that the patterns of social media consumption are influenced not only by user preferences, but also by technological changes and the availability of the platforms. For instance, social media companies may not survive and whole platforms could disappear, thus impeding data access. With changes in consumption patterns, PO may be difficult to measure consistently over time. From a more technical perspective, it is also important to assess the extent to which databases composed of social media texts collected by different means (e.g. different search queries or different platform algorithms) might raise consistency and replicability issues (e.g. [34]). As for reliability, several issues are worth considering. Even though SMD can provide complementary information to survey estimates though linkage, there are sometimes concerns about the veracity or honesty of the information collected. For instance, SMD may increase the potential for social stigmatisation, causing users to be more reluctant to share their true opinions [63]. However, the opposite may also be true: users could express more radical opinions to gain social approval (e.g. disinhibition effect). The identity of those who post can also raise veracity concerns [55], and it may be difficult to distinguish sarcastic content from texts that are straight-forwardly positive or negative (e.g. [35]). Another important issue is that we usually know how many people have liked a post, clicked on a link, or retweeted a message, but we rarely know how many people have seen the item and chosen not to take any action [77]. Furthermore, due to algorithms that favour selective exposure and homophily of opinion [6, 17], it is important to assess the extent to which findings derived from online opinion generate more polarised opinions than the ones that would be obtained through the private setting of surveys.

When researchers aim to generate new insights, they should consider that each social media platform has particular arrangements. For instance, the orientation of the content (e.g. political, family-oriented, business-oriented) as well as the scope of the content (e.g. possible bias toward more visible events) can play a decisive role on what content is available and which user profiles are most likely to be active on the social media platform. Furthermore, the nature of the platform allows for different levels of engagement in debates (e.g. Twitter is mostly used for short text content, while YouTube and Instagram allow sharing and commenting on videos and pictures). Functional capabilities can not only influence the ways of recruiting respondents for a second survey phase (e.g. direct messages), but also the identifying of sub-groups of interest (e.g. differences between friend and follower networks, and the reciprocity of follower networks). In addition, social media platforms may give users control over the availability of the information (e.g. to suppress or filter unwanted comments), which will again impact what is available from whom and on what.

For each research purpose, we should also consider that there are important ethical factors that are likely to influence the possible paths of research relying on SMD. Each platform has its own rules which are subject to change at any time. For instance, anonymity settings also affect the content of SMD, with growing concerns about surveillance and the resulting loss of privacy [29, 76, 78], thus influencing what people are willing to post. There are also evolving rules about the banning of particular words and behaviours, as well as users, which may influence research findings (especially when conducting longitudinal research). SMD are private property of tech companies and can be arbitrarily erased or made inaccessible, compromising the replicability of research.

### Outlook

Our review has several limitations. First, it focuses on social media but do not include other data sources that are frequently compared to survey data to model PO (e.g. Google trends, mainstream media, or administrative data). We thus encourage future research to extend the proposed complementary framework to additional data sources. This would allow the building of knowledge about the most suitable ways of combining these data for answering specific research purposes. Furthermore, our review entails a conceptual aim with less focus on the variety of methods used to either collect, clean, analyse, and aggregate the data to generate statistics. Discussing the pros and cons of methodologies employed by these papers could constitute the object of another review.

Notwithstanding these limitations, our study is not only of interest for social and political scientists concerned by the declining response rates and restrictive budgeting for survey research [57]. As social media have been established as multifunctional tools, and many companies and researchers implement strategies based on social media to collect opinions, make predictions, study behaviours, conduct experiments, or recruit hard-to-reach populations, this review is also of interest for practitioners.

Extracting PO from social media text can foster social sciences by moving it forward as an applied field, thus bridging gaps between computational models and interpretative research. We see this collaboration as particularly important for developing more advanced and reliable measures of opinion from social media texts. This also constitutes an opportunity to challenge the opposition of the so-called *data-driven* and *theory-driven approaches*, a simplistic dichotomy which further consolidates the misconception that social research can be conducted by relying solely on text-based data. We encourage researchers to acknowledge the different conceptualisation of opinion when measured by SMD and surveys, and we advise them to adopt a mixed-method strategy where the complementarity of both data is paramount.

## Data Availability

Please refer to the appendix.

## Appendix

**Table 2:** List of theoretical papers focusing on the combination of survey and social media data (n = 46, continues next pages)

Author(s)	Date	Title	Source	Focus
Blumenthal	2005	*Toward an open-source methodology: What we can learn from the blogosphere*	The Public Opinion Quarterly	General
Gayo-Avello	2011	*Don’t turn social media into another Literary Digest poll*	Communications of the ACM	Prediction
Sobkowicz et al.	2012	*Opinion mining in social media: Modeling, simulating, and forecasting political opinions in the web*	Government Information Quarterly	Prediction
Boyd & Crawford	2012	*Critical questions for big data: Provocations for a cultural, technological, and scholarly phenomenon*	Information, Communication & Society	General
Metaxas & Mustafaraj	2012	*Social media and the elections*	Science	Prediction
Gayo-Avello	2012	*I Wanted to Predict Elections with Twitter and all I got was this Lousy Paper*	arXiv Preprint	Prediction
Gayo-Avello	2012	*No, You Cannot Predict Elections with Twitter*	IEEE Internet Computing	Prediction
Smith	2013	*Survey-research paradigms old and new*	International Journal of Public Opinion Research	Ontology
Couper	2013	*Is the sky falling? New technology, changing media, and the future of surveys*	Survey Research Methods	General
Baker et al.	2013	*Summary Report of the AAPOR Task Force on Non-probability Sampling*	Journal of Survey Statistics and Methodology	General
Schoen et al.	2013	*The Power of Prediction with Social Media*	Internet Research	Prediction
Gayo-Avello	2013	*A Meta-Analysis of State-of-the-Art Electoral Prediction From Twitter Data*	Social Science Computer Review	Prediction
Stieglitz & Dang-Xuan	2013	*Social media and political communication: a social media analytics framework*	Social Network Analysis and Mining volume	General
Gayo-Avello et al.	2013	*Understanding the predictive power of social media*	Internet Research	Prediction
Ruths & Pfeffer	2014	*Social Media for Large Studies of Behavior*	Science	Behaviour
Tufekci	2014	*Big Questions for social media Big Data: Representativeness, Validity, and Other Methodological Pitfalls*	arXiv Preprint	Error sources
Murphy et al.	2014	*Social media, sociality, and survey research*	Book chapter (1): Social Media, Sociality, and Survey Research	Ontology
Murphy et al.	2014	*Social media in public opinion research: Executive summary of the aapor task force on emerging technologies in public opinion research*	Public Opinion Quarterly	General
Hill & Dever	2014	*The Future of Social Media, Sociality, and Survey Research*	Book chapter (12): Social Media, Sociality, and Survey Research	General
Tang et al.	2014	*Mining social media with social theories: a survey*	ACM SIGKDD Explorations Newsletter	Ontology
Zagheni & Weber	2015	*Demographic Research with Non-representative Internet Data*	International Journal of Manpower	Demographic
Resnick et al.	2015	*What social media data we are missing and how to get it*	The American Academy of Political and Social Science	General
Ampofo et ak.	2015	*Text Mining and Social Media: When Quantitative Meets Qualitative, and Software Meets Humans*	Book chapter (8): Innovations in Digital Research Methods	Ontology
Hargittai	2015	*Is Bigger Always Better? Potential Biases of Big Data Derived from Social Network Sites*	The American Academy of Political and Social Science	General
Schober et al.	2016	*Social media analyses for social measurement*	Public Opinion Quarterly	General
Olteanu et al.	2016	*Social Data: Biases, Methodological Pitfalls, and Ethical Boundaries*	Frontiers in Big Data	Error sources
Junngherr	2016	*Twitter use in election campaigns: A systematic literature review*	Journal of Information Technology & Politics	Prediction
Spiro	2016	*Research opportunities at the intersection of social media and survey data*	Current Opinion in Psychology	Linking
RJ Dalton	2016	*The potential of big data for the cross-national study of political behavior*	International Journal of Sociology	Behaviour
Johnson & Smith	2017	*Big Data and Survey Research: Supplement or Substitute?*	Book chapter: Seeing Cities Through Big Data	Linking
Hsieh & Murphy	2017	*Total Twitter error*	Book chapter (2): Total Survey Error in Practice	Error sources
Salleh	2017	*From survey to social media: Public opinion and politics in the age of big data*	Science	General
Pal	2017	*Studying political communication on Twitter: the case for small data*	Current Opinion in Behavioral Sciences	Small data
Salunkhe et al.	2017	*A review: Prediction of election using twitter sentiment analysis*	International Journal of Advanced Research in Science, Communication and Technology	Prediction
Klašnja et al.	2018	*Measuring Public Opinion with social media Data*	Book chapter: The Oxford Handbook of Polling and Survey Methods	General
Jungherr	2018	*Normalizing digital trace data*	Book chapter: Digital Discussions	General
Kwak & Cho	2018	*Analyzing public opinion with social media data during election periods: A selective literature review*	Asian Journal for Public Opinion Research	Prediction
Szreder	2018	*Will big data affect opinion polls?*	Archives of Data Science	General
Freelon	2019	*Inferring individual-level characteristics from digital trace data: Issues and recommendations*	Book chapter: Digital Discussions	Demographic
Trottier	2019	*A research agenda for social media surveillance*	Fast Capitalism	General
Sen et al.	2019	*A Total Error Framework for Digital Traces of Humans*	arXiv Preprint	Error sources
Salvatore et al.	2020	*Social Media and Twitter Data Quality for New Social Indicators*	Social Indicators Research	Error sources
Stier et al.	2020	*Integrating survey data and digital trace data: key issues in developing an emerging field*	Social Science Computer Review	Linking
Romele et al.	2020	*Digital hermeneutics: from interpreting with machines to interpretational machines*	AI & SOCIETY	Ontology
Skoric et al.	2020	*Electoral and Public Opinion Forecasts with social media Data: A Meta-Analysis*	Information	Prediction
Rousidis et al.	2020	*Social media prediction: a literature review*	Multimedia Tools and Applications	Prediction
Chauhan et al.	2020	*The emergence of social media data and sentiment analysis in election prediction*	Journal of Ambient Intelligence and Humanized Computing	Prediction

**Table 3:** List of publications combining social media and survey data for prediction purposes (n = 48, continues next pages)

Author(s)	Date	Title	Source	SM	Topic	Level of analysis
Tumasjan et al.	2011	*Election forecasts with Twitter: How 140 characters reflect the political landscape*	Social Science Computer Review	Twitter	election	national
Aparaschivei	2011	*The use of new media in electoral campaigns: Analysis on the use of blogs, Facebook, Twitter and YouTube in the 2009 Romanian presidential campaign*	Journal of Media Research-Revista de Studii Media	Twitter & Facebook & Youtube	election	national
Jungherr et al.	2012	*Why the pirate party won the German election of 2009 or the trouble with predictions: A response to Tumasjan*	Social science computer review	Twitter	election	national
Borondo et al.	2012	*Characterizing and modeling an electoral campaign in the context of Twitter: 2011 Spanish Presidential election as a case study*	Chaos An Interdisciplinary Journal of Nonlinear Science	Twitter	election	national
Jungherr et al.	2012	*Why the pirate party won the German election of 2009 or the trouble with predictions*	Social science computer review	Twitter	election	national
Choy et al.	2012	*US Presidential Election 2012 Prediction using Census Corrected Twitter Model*	arXiv Preprint	Twitter	election	national
Borondo et al.	2012	*Characterizing and modeling an electoral campaign inthe context of twitter: 2011 Spanish presidential election as a case study*	Chaos: An Interdisciplinary Journal of Nonlinear Science	Twitter	election	national
González-Bailón et al.	2012	*Emotions, Public Opinion and U.S. Presidential Approval Rates: A 5 year Analysis of Online Political Discussions*	Human Communication Research	other social media (Usenet)	presidential approval	national
Franch	2013	*(Wisdom of the Crowds)2: 2010 UK Election Prediction with S*ocial Media	Journal of Information Technology & Politics	Facebook, Twitter, Google, & YouTube	election	national
Kermanidis & Maragoudakis	2013	*Political sentiment analysis of tweets before and after the Greek elections of May 2012*	Int. J. Social Network Mining	Twitter	election	national
Fu & Chan	2013	*Analyzing online sentiment to predict telephone poll results*	Cyberpsychology, Behavior, and Social Networking	online discussion forums, personal blogs, and microblogs	election	local
DiGrazia et al.	2013	*More Tweets, More Votes: social media as a Quantitative Indicator of Political Behavior*	PloS one	Twitter	politics	district
Paul & Dredze	2014	*Discovering health topics in social media using topic models*	PloS one	Twitter	health	national
Cheng & Chen	2014	*Global* social media*, local context: A case study of Chinese-language tweets about the 2012 presidential election in Taiwan*	Aslib Journal of Information Management	Twitter	politics	regional
Ceron et al.	2014	*Every tweet counts? How sentiment analysis of social media can improve our knowledge of citizens’ political preferences with an application to Italy and France*	New media & society	Twitter	politics	national
Ceron et al.	2015	*Using sentiment analysis to monitor electoral campaigns: Method matters—evidence from the United States and Italy*	Social Science Computer Review	Twitter	election	national
Murthy	2015	*Twitter and elections: are tweets, predictive, reactive, or a form of buzz?*	Information, Communication & Society	Twitter	election	State
Eom et al.	2015	*Twitter-based analysis of the dynamics of collective attention to political parties*	PloS one	Twitter	election	national
Wong et al.	2015	*Twitter sentiment predicts Affordable Care Act marketplace enrollment*	Journal of medical internet research	Twitter	health	national
Durahim & Coşkun	2015	*# iamhappybecause: Gross National Happiness through Twitter analysis and big data*	Technological Forecasting and Social Change	Twitter	well-being	national & regional
Huberty	2015	*Can We Vote with Our Tweet? On the Perennial Difficulty of Election Forecasting with S*ocial Media	International Journal of Forecasting	Twitter	election	national
Jungherr et al.	2015	*Digital Trace Data in the Study of Public Opinion: An Indicator of Attention Toward Politics Rather Than Political Support*	Social Science Computer Review	Twitter	election	national
Burnap et al.	2016	*140 characters to victory?: Using Twitter to predict the UK 2015 General Election*	Electoral Studies	Twitter	election	national
Cody et al.	2016	*Public opinion polling with Twitter*	arXiv Preprint	Twitter	presidential approval	national
Beauchamp	2017	*Predicting and interpolating state-level polls using Twitter textual data*	American Journal of Political Science	Twitter	election	State
Yaqub et al.	2017	*Analysis of political discourse on twitter in the context of the 2016 US presidential elections*	Government Information Quarterly	Twitter	election	national
Lopez et al.	2017	*Predicting the Brexit vote by tracking and classifying public opinion using twitter data*	Statistics, Politics and Policy	Twitter	Brexit	national
Vepsäläinen et al.	2017	*Facebook likes and public opinion: Predicting the 2015 Finnish parliamentary elections*	Government Information Quarterly	Facebook	election	national
Feng et al.	2017	*Twitter analysis of California’s failed campaign to raise the state’s tobacco tax by popular vote in 2012*	Tobacco Control	Twitter	health	national
Kristensen et al.	2017	*Parsimonious data: How a single Facebook like predicts voting behavior in multiparty systems*	PloS one	Facebook	politics	national
Beauchamp	2017	*Predicting and interpolating state-level polls using Twitter textual data*	American Journal of Political Science	Twitter	election	national
Oliveira et al.	2017	*Can social media reveal the preferences of voters? A comparison between sentiment analysis and traditional opinion polls*	Journal of Information Technology & Politics	Twitter	election	national
Masoomali et al.	2018	*Using Facebook Ad Data to Track the Global Digital Gender Gap*	World Development	Facebook	equality (gender, immigration, LGB, etc.)	international
Bastos & Mercea	2018	*Parametrizing Brexit: mapping Twitter political space to parliamentary constituencies*	Information, Communication & Society	Twitter	Brexit	constituencies
Chmielewska-Szlajfer	2018	*Opinion dailies* versus *Facebook fan pages: the case of Poland’s surprising 2015 presidential elections*	Media, Culture & Society	Facebook	election	national
Pasek et al.	2018	*The stability of economic correlations over time: identifying conditions under which survey tracking polls and Twitter sentiment yield similar conclusions*	Public Opinion Quarterly	Twitter	economic satisfaction	national
Heredia et al.	2018	*S*ocial media *for polling and predicting United States election outcome*	Social Network Analysis and Mining	Twitter	election	national
Zhang	2018	*S*ocial media *popularity and election results: A study of the 2016 Taiwanese general election*	PloS one	Facebook	election	national
Bansal & Srivastava	2018	*On predicting elections with hybrid topic based sentiment analysis of tweets*	Procedia Computer Science	Twitter	election	State
Grimaldi	2019	*Can we analyse political discourse using Twitter? Evidence from Spanish 2019 presidential election*	Social Network Analysis and Mining	Twitter	election	national
Awais et al.	2019	*Leveraging big data for politics: predicting general election of Pakistan using a novel rigged model*	Journal of Ambient Intelligence and Humanized Computing	Twitter	election	national
Pasek et al.	2019	*Who’s Tweeting About the President? What Big Survey Data Can Tell Us About Digital Traces?*	Social Science Computer Review	Twitter	presidential approval	national
Jaidka et al.	2019	*Predicting elections from social media: a three-country, three-method comparative study*	Asian Journal of Communication	Twitter	election	international
Pasek et al.	2020	*Who’s tweeting about the president? What big survey data can tell us about digital traces?*	Social Science Computer Review	Twitter	presidential approval	national
Chin & Wang	2020	*A New Insight into Combining Forecasts for Elections: The Role of Social Media*	Journal of Forecasting	Facebook	election	County & city
Stieglitz et al.	2020	*Going back in time to predict the future-the complex role of the data collection period in social media analytics*	Information Systems Frontiers	Twitter	election	international
Gong et al.	2020	*Measuring relative opinion from location-based* social media*: A case study of the 2016 US presidential election*	Plos one	Twitter	election	State
Sepúlveda & Norambuena	2020	*Twitter sentiment analysis for the estimation of voting intention in the 2017 Chilean elections*	Intelligent Data Analysis	Twitter	election	national

**Table 4:** List of publications combining social media and survey data for enrichment purposes (n = 9)

Author(s)	Date	Title	Source	SM	Topic	Level of analysis
Vaccari et al.	2015	*Political expression and action on* social media*: Exploring the relationship between lower- and higher-threshold political activities among Twitter users in Italy*	Journal of Computer-Mediated Communication	Twitter	politics	national
Karlsen & Enjolras	2016	*Styles of social media campaigning and influence in a hybrid political communication system: Linking candidate survey data with Twitter data*	The International Journal of Press/Politics	Twitter	election	national
Hofstra et al.	2017	*Sources of segregation in social networks: A novel approach using Facebook*	American Sociological Review	Facebook	equality (gender, immigration, LGB, etc.)	national
Quinlan et al.	2018	*‘Show me the money and the party!’ – variation in Facebook and Twitter adoption by politicians*	Information, Communication & Society	Twitter & Facebook	political communication	national
Stier et al.	2018	*Election campaigning on* social media*: Politicians,audiences, and the mediation of political communication on Facebook and Twitter*	Political communication	Twitter & Facebook	politics	national
Cardenal et al.	2019	*Is Facebook eroding the public agenda? Evidence from survey and web-tracking data*	International Journal of Public Opinion Research	Facebook	politics	national
Jacbs & Spierings	2019	*A populist paradise? Examining populists’ Twitter adoption and use*	Information, Communication & Society	Twitter	politics	national
De Sio & Weber	2020	*Issue yield, campaign communication, and electoral performance: a six-country comparative analysis*	West European Politics	Twitter	politics	international
Shin	2020	*How Do Partisans Consume News on S*ocial Media*? A Comparison of Self-Reports With Digital Trace Measures Among Twitter Users*	Social Media + Society	Twitter	politics	national

**Table 5:** List of publications using survey as proxy with social media data (n = 18, continues next page)

Author(s)	Date	Title	Source	SM	Topic	Level of analysis
Vaccari & Nielsen	2013	*What drives politicians’ online popularity? An analysis of the 2010 US midterm elections*	Journal of Information Technology & Politics	Facebook, Twitter, and YouTube	election	national
LaMarre & Suzuki-Lambrecht	2013	*Tweeting democracy? Examining Twitter as an online public relations strategy for congressional campaigns’*	Public relations review	Twitter	election	users
Jensen & Anstead	2013	*Psephological investigations: Tweets, votes, and unknown unknowns in the Republican nomination process*	Policy & Internet	Twitter	election	State
Larsson	2015	*The EU Parliament on Twitter—Assessing the permanent online practices of parliamentarians*	Journal of Information Technology & Politics	Twitter	political communication	international
Theocharis et al.	2016	*A bad workman blames his tweets: the consequences of citizens’ uncivil Twitter use when interacting with party candidates*	Journal of Communication	Twitter	politics	national
Ceron & d’Adda	2016	*E-campaigning on Twitter: The effectiveness of distributive promises and negative campaign in the 2013 Italian election*	New media & society	Twitter	politics	national
Park et al.	2017	*Cultural values and cross-cultural video consumption on YouTube*	PLoS one	Youtube	other	international
Ernst et al.	2017	*Extreme parties and populism: an analysis of Facebook and Twitter across six countries*	Information, Communication & Society	Twitter & Facebook	politics	national
Stier et al.	2018	*Election campaigning on* social media*: Politicians, audiences, and the mediation of political communication on Facebook and Twitter*	Political communication	Twitter & Facebook	political communication	national
Barberá & Zeitzoff	2018	*The new public address system: why do world leaders adopt* social media*?*	International Studies Quarterly	Twitter & Facebook	political communication	international
Rossini et al.	2018	*The relationship between race competitiveness, standing in the polls, and social media communication strategies during the 2014 U.S. gubernatorial campaigns*	Journal of Information Technology & Politics	Twitter & Facebook	political communication	national
Rossini et al.	2018	Social Media*, Opinion Polls, and the Use of Persuasive Messages During the 2016 US Election Primaries*	Social Media + Society	Twitter & Facebook	political communication	national
Rossini et al.	2018	*S*ocial media*, opinion polls, and the use of persuasive messages during the 2016 US election primaries*	Social Media + Society	Twitter & Facebook	political communication	national
Plescia et al.	2019	*Filling the Void? Political Responsiveness of Populist Parties*	Representation	Twitter	politics	international
Wells et al.	2020	*Trump, Twitter, and news media responsiveness: A media systems approach*	New Media & Society	Twitter	politics	national
Lazarus & Thornton	2020	*Bully Pulpit? Twitter Users’ Engagement With President Trump’s Tweets*	Social Science Computer Review	Twitter	politics	national
Daniel & Obholzer	2020	*Reaching out to the voter? Campaigning on Twitter during the 2019 European elections*	Research & Politics	Twitter	political communication	international
Eberl et al.	2020	*What’s in a post? How sentiment and issue salience affect users’ emotional reactions on Facebook*	Journal of Information Technology & Politics	Facebook	politics	national

**Table 6:** List of publications combining social media and survey data for comparison purposes (n = 26, continues next pages)

Author(s)	Date	Title	Source	SM	Topic	Level of analysis
King et al.	2013	*Twitter and the health reforms in the English National Health Service*	Health policy	Twitter	health	national
Tsou et al.	2013	*Mapping social activities and concepts with social media (Twitter) and web search engines (Yahoo and Bing): a case study in 2012 US Presidential Election*	Cartography and Geographic Information Science	Twitter (+ web pages)	election	national
Kim et al.	2013	*Can tweets replace polls? A US health-care reform case study*	Book chapter (3): Social Media, Sociality, and Survey Research	Twitter	politics	national
Ceron et al.	2014	*Every tweet counts? How sentiment analysis of social media can improve our knowledge of citizens’ political preferences with an application to Italy and France*	New media & society	Twitter	election	national
Barry	2014	*Using social media to discover public values, interests, and perceptions about cattle grazing on park lands*	Environmental management	FlickrTM (pictures+comments)	climate & environment & energy	national
Van Dalen et al.	2015	*Policy considerations on Facebook: Agendas, coherence, and communication patterns in the 2011 Danish parliamentary elections*	Journal of Information Technology & Politics	Facebook	politics	national
Jungherr et al.	2016	*The mediation of politics through Twitter: An analysis of messages posted during the campaign for the German federal election 2013*	Journal of Computer-Mediated Communication	Twitter	election	national
Bhattacharya et al.	2016	*Perceptions of presidential candidates’ personalities in twitter*	Journal of the Association for Information Science and Technology	Twitter	politics	national
Diaz et al.	2017	*Online and social media Data As an Imperfect Continuous Panel Survey*	PloS one	Twitter	politics	international
Grčar et al.	2017	*Stance and influence of Twitter users regarding the Brexit referendum*	Computational social networks	Twitter	Brexit	national
Davis et al.	2017	*Public response to Obamacare on Twitter*	Journal of medical Internet research	Twitter	health	national
Heikinheimo et al.	2017	User-generated geographic information for visitor monitoring in a national park: A comparison of social media data and visitor survey	International Journal of Fgeo-Information	Instagram	other	regional
Bajaj	2017	*The use of Twitter during the 2014 Indian general elections: Framing, agenda-setting, and the personalization of politics*	Asian Survey	Twitter	political communication	national
Wang et al.	2018	*Comparing social media Data and Survey Data in Assessing the Attractiveness of Beijing Olympic Forest Park*	Sustainability	other social media	other	local
Farhadloo et al.	2018	*Associations of topics of discussion on Twitter with survey measures of attitudes, knowledge, and behaviors related to Zika: probabilistic study in the United States*	JMIR Public Health Surveillance	Twitter	health	national
Howell et al.	2018	*National Academies of Sciences, Engineering, and Medicine report on genetically engineered crops influences public discourse*	Politics and the Life Sciences	Twitter	health	national
Wainger et al.	2018	*Evidence of a shared value for nature*	Ecological Economics	Twitter	climate & environment & energy	national
Nawa et al.	2018	*Analysis of public discourse on heart transplantation in Japan using social network service data*	American Journal of Transplantation	Twitter	health	national
Scarborough	2018	*Feminist Twitter and Gender Attitudes: Opportunities and Limitations to Using Twitter in the Study of Public Opinion*	Socius	Twitter	equality (gender, immigration, LGB, etc.)	region & State & national
Mancosu & Bobba	2019	*Using deep-learning algorithms to derive basic characteristics of social media users: The Brexit campaign as a case study*	PloS one	Facebbok	politics	national
Merkley et al.	2020	*A Rare Moment of Cross-Partisan Consensus: Elite and Public Response to the COVID-19 Pandemic in Canada*	Canadian Journal of Political Science	Twitter (+GoogleTrend)	health	national
Loureiro & Alló	2020	*Sensing climate change and energy issues: Sentiment and emotion analysis with social media in the U.K. and Spain*	Energy Policy	Twitter	climate & environment & energy	international
Amaya et al.	2020	*Measuring the Strength of Attitudes in Social Media Data*	book chapter (5): Big Data Meets Survey Science: A Collection of Innovative Methods	Reddit	health	national
Klingeren et al.	2020	Public opinion on Twitter? How vote choice and arguments on Twitter comply with patterns in survey data, evidence from the 2016 Ukraine referendum in the Netherlands	Acta Politica	Twitter	politics	national
Pasek et al.	2020	*Attention to Campaign Events: Do Twitter and Self-ReportMetrics Tell the Same Story?*	book chapter (6): Big Data Meets Survey Science: A Collection of Innovative Methods	Twitter	campaign events	national
Nowak et al.	2020	Comparing covariation among vaccine hesitancy and broader beliefs within Twitter and survey data	PloS one	Twitter	health	national

**Table 7:** List of publications combining social media and survey data for generating new insights (n = 32, continues next pages)

Author(s)	Date	Title	Source	SM	Topic	Reason to complement	Level of analysis
Ampofo et al.	2011	*Trust, confidence, and credibility: Citizen responses on twitter to opinion polls during the 2010 UK general election*	Information, Communication & Society	Twitter	election	what citizens think about surveys	national
Robillard et al.	2013	*Utilizing social media to study information-seeking and ethical issues in gene therapy*	Journal of Medical Internet Research	Yahoo! Answers	health	capture emergent opinions	international
Cavazos-Rehg et al.	2014	*Characterizing the followers and tweets of a marijuana-focused Twitter handle*	Journal of Medical Internet Research	Twitter	health	capture emergent opinions	international
Russell Neuman et al.	2014	*The dynamics of public attention: Agenda-setting theory meets big data*	Journal of Communication	Twitter, blogs, forum commentaries, and traditional media news stories	politics	alternative to self-reported measures	national
Kim & Kim	2014	*Public Opinion Sensing and Trend Analysis on Social Media: A Study on Nuclear Power on Twitter*	International Journal of Multimedia and Ubiquitous Engineering	Twitter	climate & environment & energy	capture emergent opinions	national
Trilling	2015	*Two different debates? Investigating the relationship between a political debate on TV and simultaneous comments on Twitter*	Social science computer review	Twitter (+ transcript of TV debate)	election	more nuanced approach of PO	national
Williams et al.	2015	*Network analysis reveals open forums and echo chambers in social media discussions of climate change*	Global Environmental Change	Twitter	climate & environment & energy	more dynamic perspective of PO	international
Kirilenko et al.	2015	*People as sensors: Mass media and local temperature influence climate change discussion on Twitter*	Global Environmental Change	Twitter	climate & environment & energy	“passive survey” of PO	national & regional
Thompson et al.	2015	*Prevalence of marijuana-related traffic on Twitter, 2012–2013: a content analysis*	Cyberpsychology, Behavior, and Social Networking	Twitter	health	capture emergent opinions	national
Sajuria & Fábrega	2016	*Do we need polls? why Twitter will not replace opinion surveys, but can complement them*	Digital Methods for Social Science	Twitter	politics	alternative to self-reported measures	national
Settle et al.	2016	*From posting to voting: The effects of political competition on online political engagement*	Political Science Research and Methods	Facebook	politics	alternative to self-reported measures	State
Marchetti & Ceccobelli	2016	*Twitter and television in a hybrid media system: the 2013 Italian election campaign*	Journalism Practice	Twitter	election	more nuanced approach of PO	national
Krauss et al.	2017	*“Get drunk. Smoke weed. Have fun.”: a content analysis of tweets about marijuana and alcohol*	American Journal of Health Promotion	Twitter	health	capture emergent opinions	national
Barisione & Ceron	2017	*A Digital Movement of Opinion? Contesting Austerity Through S*ocial Media	Social Media and European Politics	Twitter	economic satisfaction		national
Chan & Fu	2017	*The relationship between cyberbalkanization and opinion polarization: Time-series analysis on Facebook pages and opinion polls during the Hong Kong Occupy*	Journal of Computer-Mediated Communication	Facebook	politics	more dynamic perspective of PO	city (Hong Kong)
Flores	2017	*Do anti-immigrant laws shape public sentiment? A study of Arizona’s SB 1070 using Twitter data*	American Journal of Sociology	Twitter	equality (gender, immigration, LGB, etc.)	causal inference	State
Stautz et al.	2017	*Reactions on Twitter to updated alcohol guidelines in the UK: a content analysis*	BMJ open	Twitter	health	more nuanced approach of PO	national
Chadwick & Dennis	2017	Social media*, professional media and mobilisation in contemporary Britain: Explaining the strengths and weaknesses of the Citizens’ Movement 38 Degrees*	Political Studies	Twitter (+ campaign emails & online news articles)	politics	more nuanced approach of PO	national
Etter at al.	2018	*Measuring organizational legitimacy in*social media*: Assessing citizens’ judgments with sentiment analysis*	Business & Society	Twitter	economic satisfaction	expand the scope of survey focus	national
Karami et al.	2018	*Mining public opinion about economic issues: Twitter and the us presidential election*	International Journal of Strategic Decision Sciences	Twitter	election	more nuanced approach of PO	national
Clark et al.	2018	*Using Twitter to study public discourse in the wake of judicial decisions: Public reactions to the Supreme Court’s same-sex-marriage cases*	Journal of Law and Courts	Twitter	equality (gender, immigration, LGB, etc.)	expand the scope of survey focus	national
Couper et al.	2019	*Developing a global indicator for Aichi Target 1 by merging online data sources to measure biodiversity awareness and engagement*	Biological Conservation	Twitter (+ GoogleSearches & media)	climate & environment & energy	expand the scope of survey focus	International
Aydogan et al.	2019	*Ideological congruence and social media text as data*	Journal of Representative Democracy	Twitter	politics	novel approach	national
Hatipoğlu et al.	2019	*Automated text analysis and international relations: The introduction and application of a novel technique for Twitter*	All Azimuth: A Journal of Foreign Policy and Peace	Twitter	politics	expand the scope of survey focus	national
Vidal-Alaball et al.	2019	*A New Tool for Public Health Opinion: Using Twitter Polls for Insight into Telemedicine*	JMIR Formative Research	Twitter	health	validate survey measurements	international
Barberá et al.	2019	*Who leads? Who follows? Measuring issue attention and agenda setting by legislators and the mass public using social media data*	American Political Science Review	Twitter	politics	expand the scope of survey focus	national
Dahlberg et al.	2020	*Democracy in context: using a distributional semantic model to study differences in the usage of democracy across languages and countries*	Zeitschrift für Vergleichende Politikwissenschaft	Different social media (+ online news)	politics	validate survey measurements	international
Lovari et al.	2020	*Blurred Shots: Investigating the Information Crisis Around Vaccination in Italy*	American Behavioral Scientist	Facebook	health		national
Adams-Cohen	2020	*Policy Change and Public Opinion: Measuring Shifting Political Sentiment with social media Data*	American Politics Research	Twitter	equality (gender, immigration, LGB, etc.)	causal inference	national & State
Tavoschi et al.	2020	*Twitter as a sentinel tool to monitor public opinion on vaccination: an opinion mining analysis from September 2016 to August 2017 in Italy*	Human Vaccines & Immunotherapeutics	Twitter	health	capture emergent opinions	national
Guan et al.	2020	*Chinese views of the United States: evidence from Weibo*	International Relations of the Asia-Pacific	Weibo	politics	capture emergent opinions	national
Kinra et al.	2020	*Examining the potential of textual big data for public policy decision-making on driverless cars: A case study from Denmark*	Transport Policy	Twitter	other	expand the scope of survey focus	national

**Table 8:** List of publications using social media as a recruitment tool (n = 8)

Author(s)	Date	Title	Source	SM	Topic	Level of analysis
Bekafigo & McBride	2013	*Who Tweets About Politics?: Political Participation of Twitter Users During the 2011 Gubernatorial Elections*	Social Science Computer Review	Twitter	election	State
Bode & Dalrymple	2014	*Politics in 140 characters or less: Campaign communication, network interaction, and political participation on Twitter*	Journal of Political Marketing	Twitter	politics	national
Vaccari et al.	2014	*Social media and political communication: A survey of Twitter users during the 2013 Italian general election*	Rivista Italiana di Scienza Politica	Twitter	politics	national
Vaccari et al.	2015	*Dual screening the political: Media events,* social media*, and citizen engagement*	Journal of Communication	Twitter	politics	national
Vaccari et al.	2015	*Political expression and action on* social media*: Exploring the relationship between lower-and higher-threshold political activities among Twitter users in Italy*	Journal of Computer-Mediated Communication	Twitter	politics	national
Kobayashi & Ichifuji	2015	Tweets that matter: Evidence from a randomized field experiment in Japan	Political Communication	Twitter	politics	national
Burke & Kraut	2016	The relationship between Facebook use and well-being depends on communication type and tie strength	Journal of computer-mediated communication	Facebook	well-being	international
Vaccari et al.	2016	*Of echo chambers and contrarian clubs: Exposure to political disagreement among German and Italian users of Twitter*	Social Media + Society	Twitter	politics	national
